# Relation of atherogenic lipoproteins with estimated glomerular filtration rate decline: a longitudinal study

**DOI:** 10.1186/s12882-015-0122-5

**Published:** 2015-08-04

**Authors:** Jennie Lin, Sumeet A. Khetarpal, Karen Terembula, Muredach P. Reilly, F. Perry Wilson

**Affiliations:** Renal Electrolyte and Hypertension Division, Department of Medicine, Perelman School of Medicine, University of Pennsylvania, 1 Founders, 3400 Spruce Street, Philadelphia, PA 19104 USA; Division of Translational Medicine and Human Genetics, Department of Medicine, Perelman School of Medicine, University of Pennsylvania, Philadelphia, USA; Cardiovascular Institute, Department of Medicine, Perelman School of Medicine, University of Pennsylvania, Philadelphia, USA; Section of Nephrology, Department of Medicine, Yale School of Medicine, Program of Applied Translational Research, Yale University, New Haven, USA

**Keywords:** Lp(a), ApoC-III, CKD progression, eGFR, Diabetes

## Abstract

**Background:**

Chronic kidney disease (CKD) is associated with dyslipidemia, but the role of atherogenic lipid fractions in CKD progression remains unclear. Here we assess whether baseline plasma levels of lipoprotein(a) [Lp(a)] and apolipoprotein C-III (apoC-III), causal cardiovascular (CV) risk factors being studied as therapeutic targets, are associated with decreasing estimated glomerular filtration rate (eGFR) over time.

**Methods:**

In the Penn Diabetes Heart Study (PDHS), a single-center observational cohort of type 2 diabetes patients without clinical CV disease or pre-existing CKD, we performed linear mixed effects modeling with incremental multivariable analysis to evaluate the effects of baseline plasma Lp(a) and apoC-III on the slope of eGFR over time for subjects with longitudinal data (N = 400).

**Results:**

Each two-fold higher plasma Lp(a) level was associated with an additional decline in eGFR by 0.50 mL/min/year in the fully adjusted model (p < 0.001). Baseline Lp(a) levels greater than the atherogenic cut-point of 30 mg/dL were associated with a decline in eGFR by 2.75 mL/min/year compared to 1.01 mL/min/year in subjects with baseline Lp(a) less than 30 mg/dL (p < 0.001). Although each two-fold higher apoC-III level was also associated with statistically significant decline in eGFR over time, as expected the association was attenuated after adjusting for baseline triglycerides, the key lipid intermediary regulated by apoC-III in circulation.

**Conclusions:**

Elevated baseline plasma Lp(a) levels are associated with a decrease in eGFR over time independent of race, lipid medication use, and albuminuria, whereas elevated baseline apoC-III levels are associated with eGFR decline in a triglyceride-dependent fashion.

**Electronic supplementary material:**

The online version of this article (doi:10.1186/s12882-015-0122-5) contains supplementary material, which is available to authorized users.

## Background

Chronic kidney disease (CKD), an independent risk factor for cardiovascular (CV) disease and CV mortality, is associated with dyslipidemia [1–4]. Among other derangements, the circulating lipid profile seen in CKD includes elevated levels of lipoprotein(a) [Lp(a)], a modified low-density lipoprotein particle covalently linked to the highly polymorphic apolipoprotein(a) [apo(a)], a glycoprotein of genetically-variable protein length that varies widely among individuals [5–8]. Dyslipidemia in CKD is also characterized by elevated levels of apolipoprotein C-III (apoC-III), an exchangeable apolipoprotein on lipoprotein particles that inhibits lipoprotein lipase, thereby reducing clearance of very-low-density-lipoproteins (VLDL) and the triglycerides (TGs) they carry [2, 9–18]. Both Lp(a) and apoC-III levels have recently been identified as genetic causal risk factors for CV disease, [5–7, 19–24] but their role in CKD development and progression remain unclear.

The purpose of this study was to probe whether baseline circulating Lp(a) and apoC-III levels are associated with the development of renal impairment in type 2 diabetes mellitus (T2DM) patients, a population already at increased risk for adverse CV and renal outcomes [2, 9–18]. Although aberrant concentrations of other lipid fractions are associated with CKD, we chose to focus on Lp(a) and apoC-III due to their already proven causality in CV disease. Using an observational cohort of T2DM patients without baseline clinical CV disease or CKD, we examined the relationship between estimated glomerular filtration rate (eGFR) decline and baseline concentrations of these lipoproteins. We hypothesized that higher baseline plasma Lp(a) and apoC-III levels are each associated with eGFR decline.

## Methods

### Study population

The Penn Diabetes Heart Study (PDHS), as previously described, [25–29] is an observational cohort of 2118 patients with T2DM, enrolled and recruited from outpatient clinics affiliated with the Hospital of the University of Pennsylvania (HUP) between 2001 and 2011. The University of Pennsylvania (Penn) Institutional Review Board approved the study protocol, and all subjects gave written informed consent. Inclusion criteria were: 1) a clinical diagnosis of T2DM (defined as fasting blood glucose >126 mg/dl, 2-hour post-prandial glucose >200 mg/dl, or use of oral hypoglycemic agents/insulin in a subject greater than age 40 years); 2) age of 35–75 years; and 3) a negative pregnancy test if female and of child-bearing age. Exclusion criteria were: 1) history of clinical CV disease defined by myocardial infarction (MI), documented angiographic coronary artery disease, positive stress test, coronary or peripheral revascularization, stroke, or transient ischemic attack; 2) insulin use prior to age 35; 3) renal insufficiency defined at the time of recruitment as serum creatinine greater than 2.5 mg/dL; 4) active infection or malignancy; and 5) weight more than 300 pounds. For the current study, subjects were additionally excluded if they did not have longitudinal serum creatinine data in the HUP electronic medical records or had baseline eGFR less than 60 mL/min/1.73 m^2^, leaving a final sample size of 400 subjects.

### Data collection

Study subjects were evaluated at the Clinical and Translational Research Center (CTRC) at HUP after a 12-hour fast. They completed a questionnaire regarding past medical, social, and family history as well as use of medications. Height, weight, waist and hip circumference, and bilateral resting systolic and diastolic blood pressures were measured. Samples of whole blood and urine were collected, processed, and stored (at −80 °C). All lipid data used in the analysis were collected at this baseline study visit. Complete blood count, basic metabolic panel, hemoglobin A1c, and albuminuria assays were performed at the clinical laboratories of HUP. TGs were measured enzymatically, while LDL-C was measured directly, after ultracentrifugation (β-centrifugation technique) in a Centers for Disease Control (CDC) certified lipid laboratory [25, 26, 28]. Lp(a) and apolipoproteins were measured by immunoturbidimetric assay (Wako Chemicals, U.S.A. Inc., Richmond, VA) on a Hitachi 912 autoanalyzer (Roche Diagnostics, Basel, Switzerland). Laboratory test results were generated by personnel blinded to the clinical characteristics of the study participants.

In this study, hypertension was defined as meeting one of the following criteria: 1) self-reported history of hypertension; 2) use of anti-hypertensive medications; 3) documented systolic blood pressure measurement greater than 140 mmHg; or 4) documented diastolic blood pressure measurement greater than 90 mmHg. eGFR was calculated based on the CKD-EPI equation [30]. Fasting homeostasis model assessment-estimated insulin resistance (HOMA-IR) was calculated for a subset of subjects using the following equation: (glucose [mg/dL] x insulin [μIU/mL] / 405) [31]. Urinary ACR for each subject, reported in mg/g, was calculated by dividing spot urine albumin by spot urine creatinine concentrations. Follow-up clinical laboratory data was available from 2001–2013, and was collected as part of routine patient care.

### Primary outcomes

Our primary outcomes of interest were the association of 1) baseline Lp(a) concentration and 2) baseline apoC-III concentration with eGFR slope. As apoC-III has a significant role in TG metabolism, [10] we additionally examined the association of baseline TG concentration with eGFR slope with and without adjustment for apoC-III concentrations and the converse of apoC-III association with eGFR slope with and without adjustment for TGs.

### Statistical analysis

For descriptive data, distributions are reported as median and interquartile range (IQR) for continuous variables and as proportions for categorical variables. Due to their skewed distributions, for multivariable analysis Lp(a) and apoC-III were log-transformed (log base 2) when analyzed as continuous variables. Linear mixed effects modeling of eGFR with the lipoprotein of interest was performed in incremental models, and the coefficient determined from multivariate mixed effects analysis represented eGFR slope. Model 1 was adjusted for age, race, gender, and baseline serum creatinine. Model 2 was also adjusted for known risk factors for CKD progression and for potential lipid-related confounders; these additional co-variates included body mass index (BMI), hypertension, use of lipid-lowering medications, smoking status, alcohol use, and hemoglobin A1c. Model 3 further adjusted for baseline urinary ACR, which was examined separately due to its potential role in affecting lipid metabolism. For additional apoC-III analysis, TGs were included in an additional model (Model 4) as a covariate due to the known biological effect of apoC-III on TG metabolism [10] i.e., we examined the impact of TGs as an intermediate effector of apoC-III on slope of eGFR. With the exception of Model 4, each step in multivariate analysis did not adjust for other lipid fractions. Additional sensitivity analysis was performed adjusting for HOMA-IR within a subset of subjects who had insulin resistance measured. Models included a random intercept term and an independent covariance structure. Model goodness-of-fit was assessed with the Akaike information criterion [32]. Statistical analyses were performed using STATA version 13.0 software (Stata Corps, College Station, TX).

## Results

### Baseline patient characteristics

Of the 2118 participants in the originally recruited cohort, 400 had follow-up creatinine data and were included in this analysis. Baseline characteristics of these 400 subjects are presented in Table 1. Except in gender distribution, these did not differ significantly from the cohort baseline characteristics as a whole (Table S1). The study participants are evenly divided between men (49 %) and women, and 36 % self-identified as Black. The majority met hypertension criteria (73 %). The median baseline eGFR was above 90 mL/min/1.73 m^2^, and the median urinary ACR was below 6 mg/g (Table 1). The median baseline Lp(a) concentrations was below the atherogenic cutpoint of 30 mg/dL, [33] and the median apoC-III concentration was below 15 mg/dL [10] (Table 1).

**Table 1:** Patient baseline characteristics

	Total (N = 400)
Age	58 (52, 63)
Gender (%)	
Men	49
Women	51
Race (%)	
White	58
Black	36
Other	6
Hypertension (%)	73
Systolic BP (mmHg)	128 (118, 136)
Diastolic BP (mmHg)	75 (70, 80)
Waist Circumference (in)	42 (38, 46)
BMI (kg/m^2^)	31.7 (28.0, 36.2)
Glucose (mg/dL)	112 (95, 139)
Hemoglobin A1c (%)	6.7 (6.2, 7.5)
Serum Creatinine (mg/dL)	0.88 (0.70, 1.0)
BUN (mg/dL)	16 (13, 19)
eGFR (CKD-EPI)	91.7 (80.0, 102.1)
Urinary ACR (mg/g)	5.3 (3, 13.5)
Anti-Hypertensive Medications (%)	
ACE Inhibitor or ARB	61
Diuretic	32
CCB	16
Beta Blocker	12
Lipid-Lowering Medications (%)	
Statin	52
Fibrate	7
Niacin	4
Lp(a) (mg/dL)	25 (9, 57)
ApoC-III (mg/dL)*	11.4 (8.7, 15.6)
Triglycerides (mg/dL)	105 (79, 156)
LDL-C (mg/dL)	99 (79, 119)
Apolipoprotein B (mg/dL)	80 (69, 94)

Characteristics of cohort at baseline visit. All data reported as median (interquartile range) unless otherwise specified

*BP* blood pressure, *BMI* body mass index, *BUN* blood urea nitrogen, *eGFR* estimated glomerular filtration rate, *ACR* albumin to creatinine ratio, *ARB* angiotensin receptor blocker, *CCB* calcium channel blocker, Lp(a) lipoprotein(a), ApoC-III apolipoprotein C-III, LDL-C low density lipoprotein cholesterol

*N = 336

### Longitudinal renal measurements

In this cohort sample, mean eGFR decline was 1.49 mL/min/1.73 m^2^ per year (95 % CI 1.31, 1.66). Among these participants, 15 % eventually progressed to CKD with decline in eGFR to less than 60 mL/min/1.73 m^2^ within 5 years. Median follow-up time was 4.5 years, and the median number of follow-up serum creatinine values per subject was 4.48 laboratory tests.

### Association between baseline Lp(a) levels and eGFR decline

**Fig. 1:**
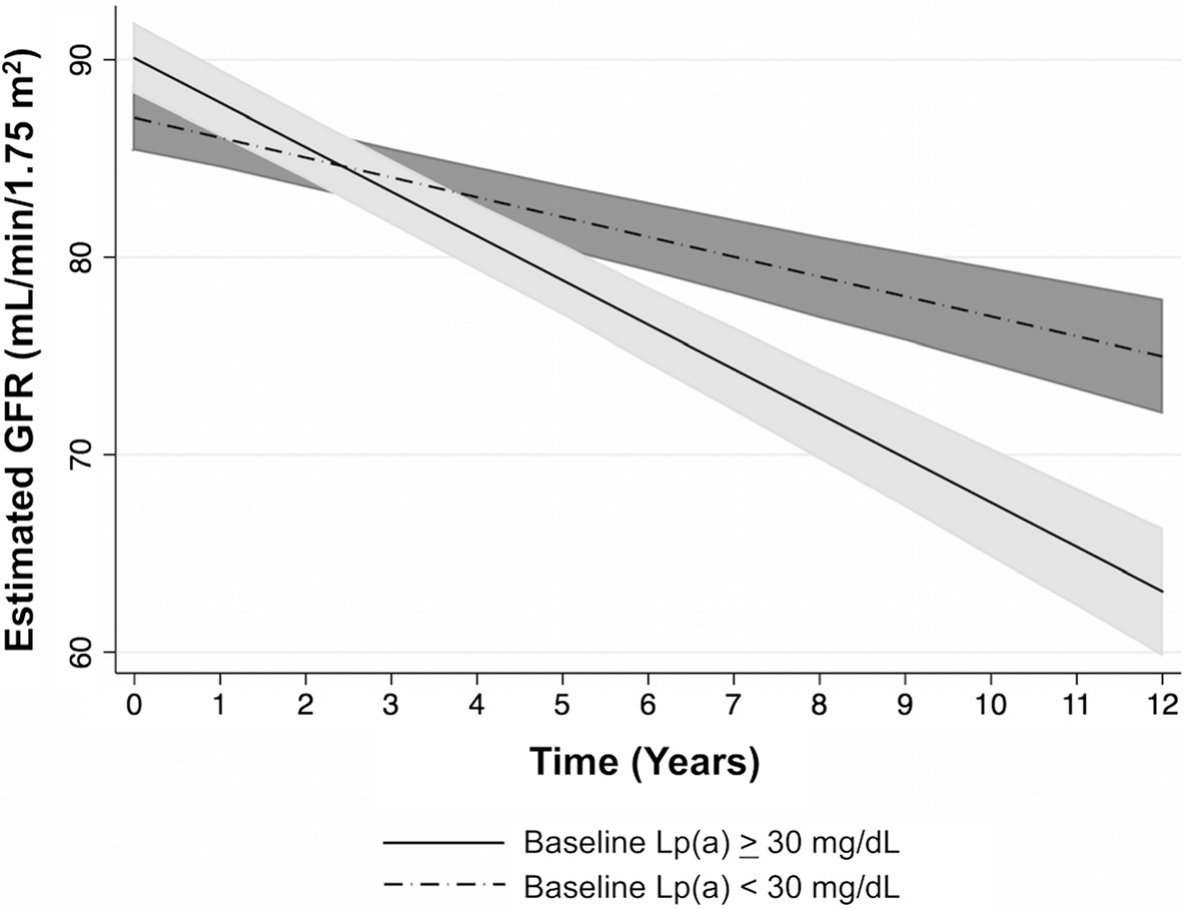
eGFR decline by baseline Lp(a) level. Baseline Lp(a) levels were divided into two groups using the atherogenic cutpoint of 30 mg/dL (baseline Lp(a) ≥ 30 N = 181; baseline Lp(a) < 30 N = 219). Regression through mixed effects modeling was performed, and graphical representation of eGFR changes over time reveals different rates of decline between the two groups. The data depicted reflect the fully adjusted models accounting for the following co-variates: age, gender, race, baseline SCr, BMI, hypertension, lipid-lowering medications, smoking, alcohol use, hemoglobin A1c, urinary ACR. Definition of hypertension includes the use of anti-hypertensive medications. Abbreviations: SCr serum creatinine, ACR albumin to creatinine ratio

Across all incremental levels of multivariable analysis, mixed effects modeling demonstrated that baseline Lp(a) concentrations had a robust association with eGFR decline (Table 2, Additional file 1: Table S1). After adjustment for age, race, gender, and baseline serum creatinine (Model 1), each two-fold increase in baseline plasma Lp(a) level was associated with a decrease in eGFR by 0.83 mL/min/1.73 m^2^ (P < 0.001) per year. A similar trend was seen in the fully adjusted model, with each two-fold higher plasma Lp(a) level holding a strong association with a decrease in eGFR by 0.50 mL/min/1.73 m^2^ (P < 0.001) per year after adjusting for demographic factors, baseline serum creatinine, BMI, hypertension, lipid-lowering medications, smoking status, alcohol use, hemoglobin A1c, and urinary ACR (Model 3). When baseline plasma Lp(a) levels were divided according to the atherogenic cutpoint of 30 mg/dL, the degree of eGFR decline was more pronounced in the >30 mg/dl group than those with Lp(a) <30 mg/dl (Fig. 1). In fully adjusted analysis, baseline Lp(a) concentrations greater than 30 mg/dL were associated with a decline in eGFR by 2.75 mL/min/1.73 m^2^ per year (95 % CI −3.11, −2.39), whereas baseline Lp(a) concentrations less than 30 mg/dL were associated with an eGFR decline of 1.01 mL/min/1.73 m^2^ (95 % CI −1.21, −0.81; p for interaction between groups <0.001) (Fig. 1, Additional file 1: Table S1).

**Table 2:** Association between two-fold higher baseline plasma Lp(a) levels and eGFR decline

Analysis	eGFR decline, ml/min/1.73 m^2^ (95 % CI)	P-Value
Model 1	−0.83 (−0.97, −0.70)	<0.001
Model 2	−0.51 (−0.65, −0.37)	<0.001
Model 3	−0.50 (−0.64, −0.36)	<0.001

Data represent eGFR change per year for every two-fold higher plasma Lp(a) concentration, analyzed as a continuous variable. Regression through mixed effects modeling was performed in incremental models with the following co-variates

Model 1: Age, gender, race, baseline SCr

Model 2: Age, gender, race, baseline SCr, BMI, hypertension, lipid-lowering medications, smoking, alcohol use, hemoglobin A1c

Model 3: Age, gender, race, baseline SCr, BMI, hypertension, lipid-lowering medications, smoking, alcohol use, hemoglobin A1c, urinary ACR

*Definition of hypertension includes the use of anti-hypertensive medications

*SCr* serum creatinine, *ACR* albumin to creatinine ratio

### Association between baseline apoC-III levels and eGFR decline

Adjusting for demographic factors only (Model 1), mixed effects modeling showed a significant association between each two-fold higher baseline apoC-III levels and a decrease in eGFR by 1.62 mL/min/1.73 m^2^ (P <0.001) per year (Table 3, Additional file 1: Table S1). This association, although mildly attenuated, held even after additionally adjusting for baseline serum creatinine, BMI, hypertension, use of lipid-lowering medications, smoking status, alcohol use, hemoglobin A1c, and albuminuria (Model 3). However, the association was markedly attenuated in the fully adjusted model, which included additional adjustment for baseline TG levels (Model 4) (slope of eGFR decline 0.22, P = 0.26). In a smaller subset of PDHS patients who had insulin resistance measured, adjustment for HOMA-IR yielded similar results (slope of eGFR decline -.17, P = 0.379). Baseline TG levels correlate with baseline apoC-III levels (Additional file 2: Table S2), consistent with the concept that these lipid factors may converge in downstream pathways.

**Table 3:** Association between Two-fold Higher Baseline ApoC-III and eGFR Decline

Analysis	eGFR decline, ml/min/1.73 m^2^ (95 % CI)	P-Value
Model 1	−1.62 (−1.93, −1.31)	<0.001
Model 2	−0.46 (−0.80, −0.13)	0.007
Model 3	−0.38 (−0.72, −0.05)	0.026
Model 4	−0.22 (−0.60, 0.16)	0.257

Data represent eGFR change per year for every two-fold increase of log-transformed plasma apoC-III concentration, analyzed as a continuous variable. Linear regression through mixed effects modeling was performed in incremental models with the following co-variates

Model 1: Age, gender, race, baseline SCr

Model 2: Age, gender, race, baseline SCr, BMI, hypertension, lipid-lowering medications, smoking, alcohol use, hemoglobin A1c

Model 3: Age, gender, race, baseline SCr, BMI, hypertension, lipid-lowering medications, smoking, alcohol use, hemoglobin A1c, urinary ACR

Model 4: Age, gender, race, baseline SCr, BMI, hypertension, lipid-lowering medications, smoking, alcohol use, hemoglobin A1c, urinary ACR, triglyceride levels

*Definition of hypertension includes the use of anti-hypertensive medications

*SCr* serum creatinine, *ACR* albumin to creatinine ratio

## Discussion

In this study of individuals with T2DM, we demonstrate that baseline plasma Lp(a) levels strongly associate with eGFR decline and that a significant although more modest association exists between baseline plasma apoC-III levels and eGFR decrease over time in a TG-dependent fashion. To our knowledge, this study is among the first to demonstrate an independent association between baseline circulating Lp(a) levels and eGFR decline in a population without pre-existing CKD.

Several prior studies have shown that plasma Lp(a) levels are elevated in CKD patients, [7, 34–36] but the direction of causality in this trend has been unclear. Past studies have explored whether Lp(a) elevations in CKD are due to reduced renal clearance, pointing to differences in Lp(a) concentrations in the renal arteriovenous circulation [37] as well as reduction in Lp(a) levels after renal transplant [38]. Although the kidney may play a role in Lp(a) metabolism, the current study introduces the possibility that circulating Lp(a) may also play a role in renal impairment. Here we show that in diabetics without baseline CKD, higher baseline Lp(a) concentrations, including above the clinical used atherogenic cutpoint of 30 mg/dL, are strongly associated with decline in eGFR. The eGFR slope for subjects Lp(a) levels above that cutpoint is more than twice as steep as the slope for subjects in the lower Lp(a) group, indicating that higher baseline Lp(a) levels may accelerate the rate of decline in eGFR that would otherwise take place due to age and the natural course of diabetic disease. Because circulating Lp(a) levels do not vary much over time within healthy individuals, [39] our results suggest that exposure to higher baseline Lp(a) concentrations that would remain unchanged over time may be a risk factor for eGFR decline. Within the PDHS cohort, the strong dose-dependent, temporal relationship between baseline plasma Lp(a) concentration and negative eGFR slope is consistent with a potential causal relationship between this lipoprotein risk factor and renal outcome. However, as has been performed for CVD outcomes, [5, 6, 20, 40, 41] genetic data and Mendelian randomization studies will be important to definitively address Lp(a) causality in renal disease in humans.

Our findings add valuable insight to the conflicting results of prior studies examining the relationship between Lp(a) and GFR. In 2005, Song *et al*. found in a small cohort of 81 individuals with proteinuric diabetic nephropathy that each 10 mg/dL increase in log-transformed Lp(a) concentration was associated with a 1.4 increased odds of serum creatinine doubling independent of albuminuria, hypertension, and glycemic control [42]. Our results complement Song *et al*.’s finding as we further demonstrate an independent inverse relationship between Lp(a) concentration and decline renal function in diabetics without baseline CKD, suggesting that Lp(a) is not only involved in progression of renal disease but also possibly the development of GFR impairment.

However, also in 2005, Uhlig *et al*. found no association between Lp(a) and GFR after adjusting for age, sex, and race in the Modification of Diet in Renal Disease (MDRD) cohort of 804 participants [35]. The MDRD study was cross-sectional in nature and focused on non-diabetic patients with pre-existing moderate CKD (iothalamate-determined GFR between 13 and 55 mL/min/1.73 m^2^), a population with different baseline characteristics from the PDHS cohort of diabetic individuals without baseline CKD. Considering these differences in study population, the cross-sectional findings of Uhlig’s study are not directly comparable to our cohort. Furthermore, over half of the participants in the MDRD cohort had renal disease of polycystic or glomerular origin and thus had other competing pathophysiologic mechanisms driving their GFR status.

In a more recent prospective study utilizing the Chronic Renal Insufficiency Cohort (CRIC), Rahman *et al*. also did not find an association between baseline plasma Lp(a) levels and their endpoints of 50 % decline in eGFR or progression to ESRD [43]. They did not find a statistically significant association between Lp(a) and any degree of eGFR decline in their fully adjusted analysis, which included the covariates of age, sex, race, diabetes status, blood pressure, statin use, smoking, proteinuria, BMI, and alcohol use. The CRIC study may suggest that Lp(a) does not predict progression of CKD, but it does not negate our findings as its baseline population characteristics are quite different. Like the MDRD cohort, CRIC consists of a patient population (both diabetics and non-diabetics) with pre-existing moderate to severe CKD. Our results suggest that Lp(a) plays a role in the early development of CKD. After CKD has developed, other more dominant pathologies (such as hyperfiltration and fibrosis) may drive subsequent eGFR decline.

Adding to previous knowledge that, like Lp(a), apoC-III is elevated in CKD, [2, 13, 15, 36, 44] here we report apoC-III’s association with eGFR decline in a TG-dependent manner. A prior small study did not find an association between apoC-III and eGFR slope, but again it utilized a very different study population consisting of 73 non-diabetic adults who had primary renal disease due to glomerular disease, polycystic kidney disease, and interstitial nephritis [15]. Our findings demonstrate a statistically significant association between baseline apoC-III levels and eGFR decline that was attenuated only partly after adjusting for known risk factors of CKD progression including hemoglobin A1c, a confounder in both CKD progression and in the lipid metabolism pathway as hyperglycemia drives TG and VLDL production [10]. As expected, the association between baseline apoC-III levels and eGFR slope was then strongly attenuated after additional adjustment for baseline TGs; this result did not change when a subset of subjects with HOMA-IR data underwent further adjustment for HOMA-IR. This result is consistent with other studies showing that TGs are a well-established intermediate of the apoC-III pathway in causing CVD, [10, 13, 45, 46] as VLDL-associated apoC-III plays a role in TG lipolysis. Although insulin resistance, which contributes to increased VLDL secretion and decreased catabolism of TG rich lipoproteins, would be an expected confounder in the interplay between apoC-III and TGs in diabetics, adjustment for it did not change our results. Further work, including Mendelian randomization strategies, will be needed to investigate whether a direct relationship between VLDL-apoC-III and eGFR decline exists and the renal mechanisms of this effect, if present.

## Conclusions

The current study has several strengths. First, the PDHS sub-sample with longitudinal eGFR data is similar to the overall PDHS cohort, is well-characterized and representative of the broader T2DM population, and thus is potentially generalizable among non-insulin-dependent diabetics. Second, the study utilizes a cohort without baseline CKD but with longitudinal follow-up, allowing for the study of potential development of early CKD. This complements other study cohorts that focus on individuals who already have pre-existing CKD. Third, our study has a unique focus of leveraging CV biomarkers measured in the PDHS cohort for the study of CKD risk as well as CVD risk.

Our study also has limitations worth consideration. Within the larger full PDHS cohort, the sample with longitudinal follow-up is considerably smaller, although the sample size was large enough to power the multivariable analysis employed in our study. We only included individuals in our study who continued to receive care at our institution and who had follow-up serum creatinine values. This could lead to selection bias, if individuals with higher levels of apoC-III or Lp(a) were more likely to seek medical care and, due to greater degrees of observation, more likely to be detected to have declining eGFR. Also, while genotypic data was available for a portion of the larger PDHS cohort, our final sample size precluded a robust Mendelian randomization study of the rare or low-frequency *LPA* and *APOC3* genetic variants and renal outcomes. As studies on *LPA* and *APOC3* variants have established a causal link between their respective lipoprotein biomarker and increased risk of CV disease, [5, 6, 20, 22, 23] leveraging genetic data as an instrumental variable for CKD risk would more fully address reverse causation and exclude other confounders. In addition, prior studies have established an inverse correlation between the size of the apo(a) portion of Lp(a) and circulating Lp(a) concentrations; [5, 19, 20] but because the Lp(a) assay used in this current study is not sensitive to apolipoprotein(a) isoform size, we are not able to establish whether the observed association between baseline Lp(a) and eGFR decline is isoform dependent. Our focus is on CKD within a T2DM setting so our findings cannot be extrapolated to non-T2DM settings. Other study limitations include the use of eGFR rather than measured GFR or cystatin-C and recruitment of patients from a single geographical area.

In summary, our data show that higher circulating baseline Lp(a) levels have a robust association with eGFR decline and that higher baseline apoC-III levels have a similar, but more modest relationship. These findings may have implications for whether Lp(a) and apoC-III are possible therapeutic targets for CKD prevention in the diabetic population. With emerging Lp(a)-lowering therapies and *APOC3* anti-sense oligonucleotides targeting apoC-III on the horizon, [47, 48] further prospective studies, including Mendelian randomization, of Lp(a) and apoC-III in larger cohorts are needed to determine causality and assess the prospects for future clinical trials.

## Additional files

Additional file 1: Table S1. PDHS Dataset: This is the primary dataset from which statistical analyses were run to generate the data of this manuscript. (XLS 1658 kb) Additional File 2: Table S2. Correlation Matrix: This is a correlation matrix of lipid fractions and lipoproteins examined in the manuscript. (PDF 34 kb)

## Abbreviations

ACR: Albumin to creatinine ratio
apo(a): Apolipoprotein(a)
apoC-III: Apolipoprotein C-III
CDC: Center for Disease Control
CKD: Chronic kidney disease
CRIC: Chronic Renal Insufficiency Cohort
CTRC: Clinical and Translational Research Center
CV: Cardiovascular
eGFR: Estimated glomerular filtration rate
ESRD: End stage renal disease
HOMA-IR: Homeostasis model assessment-estimated insulin resistance
HUP: Hospital of the University of Pennsylvania
IQR: Interquartile range
Lp(a): Lipoprotein(a)
MDRD: Modification of Diet in Renal Disease
PDHS: Penn Diabetes Heart Study
Penn: University of Pennsylvania
T2DM: Type 2 diabetes mellitus
TG: Triglyceride
VLDL: Very-low-density lipoprotein
