# Supplementary material for: Relation of atherogenic lipoproteins with estimated glomerular filtration rate decline: a longitudinal study
Source: BMC Nephrol. 2015 Aug 4;16:130. doi: 10.1186/s12882-015-0122-5 (PMC4545861; doi:10.1186/s12882-015-0122-5)
Supplement: Additional File 2: Table S2. — Correlation Matrix: This is a correlation matrix of lipid fractions and lipoproteins examined in the manuscript. (PDF 34 kb) [file 12882_2015_122_MOESM2_ESM.pdf]

Additional File 2

	Lp(a)	apoC-III	TG	LDL-C
**Lp(a)**	1.00			
**apoC-III**	0.01*	1.00		
**TG**	-0.03	0.56	1.00	
**LDL-C**	0.21	0.23	0.16	1.00

Correlation matrix of lipoproteins and lipid fractions examined. All correlations with p<0.001 except if indicated by *.
Abbreviations: Lp(a) lipoprotein(a), apoC-III apolipoprotein C-III, TG triglycerides, LDL-C low density lipoprotein cholesterol, apoB apolipoprotein B.
